# Commentary: Blood Eosinophilia Is Associated with Unfavorable Hospitalization Outcomes in Children with Bronchiolitis

**DOI:** 10.3389/fped.2016.00123

**Published:** 2016-11-21

**Authors:** Amrita Dosanjh

**Affiliations:** ^1^UCSD Medical Center, San Diego, CA, USA

**Keywords:** respiratory, respiratory syncytial virus infections, eosinophils, pediatrics, cystic fibrosis

The recently published article by Shein et al. identified 1356 inpatients aged <24 months with bronchiolitis and studied the presence of eosinophils among those patients with a complete blood count. The cohort who were eosinophil-positive were found to have more prolonged hospitalization compared to the eosinophil-negative group, OR 1.88, *p* = 0.020. Interestingly, clinical severity as described by use of mechanical ventilation was also greater in this cohort (1).

Eosinophils and eosinophil degranulation products, such as eosinophil cationic protein (ECP), have been identified in a subset of patients with bronchiolitis (2). Bronchiolitis is most commonly caused by a viral infection of the lower respiratory tract by respiratory syncytial virus (RSV). Early responses to infection of the epithelium include a neutrophilic and mononuclear cellular infiltration of the lining of the bronchioles (3, 4). Some patients exhibit both peripheral and localized eosinophilia. Eosinophils have been identified in lung biopsies in severe RSV (5).

One peripheral biomarker that may be useful in the classification of cellular responses to RSV lower respiratory tract infection is ECP. The presence of elevated serum levels of ECP is also present in other obstructive lung diseases. In a study (CHOC/Stanford) of serum samples from cystic fibrosis (CF) patients and asthmatic and bronchiolitic patients, all demonstrated elevated serum levels compared to control. The CF group had the highest ECP serum levels compared to controls and non CF patients (*p* = 0.008). In this study, ECP did neither directly correlate with IgE nor absolute eosinophil counts (6).

This suggests that there may be a subset of patients with activation of eosinophils and normal eosinophil counts, who may experience deleterious symptoms as a result of the direct cellular injury caused by ECP. Future studies may be completed to further analyze this sub-population of patients.

## Author Contributions

The author confirms being the sole contributor of this work and approved it for publication.

## Conflict of Interest Statement

The author declares that the research was conducted in the absence of any commercial or financial relationships that could be construed as a potential conflict of interest.
